# Magnetorelaxometry in the Presence of a DC Bias Field of Ferromagnetic Nanoparticles Bearing a Viscoelastic Corona

**DOI:** 10.3390/s18051661

**Published:** 2018-05-22

**Authors:** Victor Rusakov, Yuriy Raikher

**Affiliations:** Institute of Continuous Media Mechanics, Russian Academy of Sciences, Ural Branch, Perm 614013, Russia; vvr@icmm.ru

**Keywords:** magnetic nanoparticles, viscoelasticity, magnetorelaxometry

## Abstract

With allowance for orientational Brownian motion, the magnetorelaxometry (MRX) signal, i.e., the decay of magnetization generated by an ensemble of ferromagnet nanoparticles, each of which bears a macromolecular corona (a loose layer of polymer gel) is studied. The rheology of corona is modelled by the Jeffreys scheme. The latter, although comprising only three phenomenological parameters, enables one to describe a wide spectrum of viscoelastic media: from linearly viscous liquids to weakly-fluent gels. The “transverse” configuration of MRX is considered where the system is subjected to a DC (constant bias) field, whereas the probing field is applied perpendicularly to the bias one. The analysis shows that the rate of magnetization decay strongly depends on the state of corona and slows down with enhancement of the corona elasticity. In addition, for the case of “transverse” MRX, we consider the integral time, i.e., the characteristic that is applicable to relaxation processes with an arbitrary number of decay modes. Expressions for the dependence of the integral time on the corona elasticity parameter and temperature are derived.

## 1. Introduction: Magnetorelaxometry in Linear Approximation

Magnetic nanoparticles are successfully used in diverse bioengineering and medical applications both as themselves (e.g., as contrasting agents in Magnetic Resonance Imaging (MRI)) or as essential components of physicochemical complexes (magnetic polymerosomes [1,2], microferrogels [3]). These techniques span from object-oriented drug delivery [4] to active thermal action on malignant cells (magneto-inductive hyperthermia [5]) or forced penetration through cell membranes [6].

Nowadays, one of the most developed applications of magnetic nanoparticles is their introduction as sensors for diagnosing the state and content of complex media both of non-organic and biological origin. In the context of conventional microrheology approach, it is assumed that the particles do not chemically react with the medium under study and, thus, serve as the means of specific “nondestructive testing”. On the contrary, in biochemical analysis, the nanoparticles are functionalized, and the main interest is focused on the degree of reaction between the molecules of tested solution with the markers grafted to the particles or with their bare but chemically active surface. The macromolecular coating that a particle acquires as a result of this adsorption is often termed as a protein corona [7].

A unique advantage of magnetic nanosensors is that, with the aid of an applied magnetic field, one is able to remotely excite their motion and analyze the generated response to that.

The chemical processes resulting in formation of the corona on the particle surface affect its dynamic behavior. Those changes could be sensed by either measuring the magnetic spectrum of the system (if the probing is done with an AC field) or—in pulse regime—by registering the signal of the magnetization decay. The latter experimental technique is known as magnetorelaxometry (MRX). In many cases, MRX as a laboratory test is more preferable in comparison with magnetic spectroscopy, in particular, due to its much easier technical implementation [8,9,10].

Consider a ferromagnet nanoparticle that, due to its smallness, is single-domain and, thus, bears a magnetic moment $\vec{\mu }$ of constant magnitude. This particle floats in a water solution of macromolecules some of which, possessing affinity to the particle surface, adsorb on it, so that the particle environment transforms from a Newtonian liquid into a viscoelastic polymer gel.

For simplicity of the following considerations, we assume that vector $\vec{\mu }$ is “frozen” in the particle body and treat the nanoparticle as a miniature permanent magnet. This means neglecting the superparamagnetic effect: thermal fluctuations of $\vec{\mu }$ inside the particle. Such an approach implies that the Néel (internal) relaxation time of $\vec{\mu }$ is much greater than any other reference time of the problem. Certainly, this approximation is not universally valid. However, as it follows from a number of works on the subject, see, for example, reviews [11,12] for general considerations and papers [13,14] for realistic examples. The particles of 15–20 nm in diameter made of a moderately magnetically hard substance (e.g., cobalt ferrite) matches this requirement fairly well in the frequency range below 1 MHz, which is most relevant for MRX.

For the above-described magnetically hard nanoparticle suspended in a fluid medium, the only fluctuational process affecting vector $\vec{\mu }$ is its rotary Brownian motion together with the particle. Due to that, in the absence of field, the directions of magnetic moments are distributed at random, and the net magnetization of the ensemble is zero. An MRX measurement begins with application of a uniform magnetic field that orients the particle magnetic moments, thus inducing a non-zero equilibrium magnetization of the system. Then, the magnitude (or direction) of the field is abruptly changed, and the signal generated by the magnetization evolving to a new equilibrium is registered for further analysis. By that, MRX not only accomplishes its general purpose—to evaluate the amount of particles (that is proportional to the signal intensity)—but delivers information on the details of the particle rotary motion.

If the exerted field is turned off completely, the magnetization of the particle ensemble decays freely down to zero, and this process is governed solely by the rotary Brownian diffusion. There are no external factors. If the field is switched between two finite values—from $\vec{H}$ to $\vec{H}+{\vec{H}}_{1}$—the same occurs to the system magnetization. Under those conditions, the MRX signal becomes a function of both values ($\vec{H}$ and ${\vec{H}}_{1}$), by variation of which one can extract more information from the same experiment than in the case where the field is just turned off. A convenient way to study this relaxation is to change the bias field in small (∼$H_{1}\ll H$) steps to be able to use the linear response theory. In that case, the obtained MRX signal depends parametrically only on that value *H* of the field, around which the variation is done. The “linear” MRX of that kind is the subject of the present work.

## 2. Model

**Figure 1:**
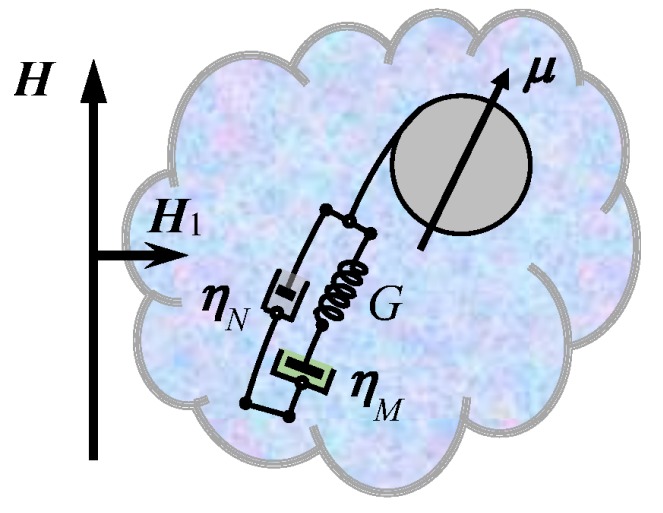
Sketch of a magnetic particle bearing a corona of Jeffreys medium. The system is set under constant field $\vec{H}$, and the existing equilibrium is perturbed by the field ${\vec{H}}_{1}$ switched on/off stepwise or alternating harmonically.

We consider a nanoparticle that, as a result of formation of a corona, dwells in a viscoelastic environment, i.e., a medium with retarded response. As a rheological model for the latter, the Jeffreys scheme (see [15], for example) is used because, unlike the plain Maxwell one, it is robust when applied to Brownian motion and is free of artifacts [16,17,18]. The viscoelastic properties of the Jeffreys model are fully rendered by three parameters (see the scheme outlined in Figure 1). A single-element chain (narrow damper) associated with the solvent there—a Newtonian fluid with the viscosity coefficient $\eta_{N}$—is set in parallel with a two-element Maxwell chain associated with the corona. The latter is assumed to possess both elasticity with modulus *G* (the spring) and intrinsic viscosity $\eta_{M}$ (wide damper). Evidently, in a medium with a pronounced visoelasticty, the Jeffreys viscosity coefficients are substantially different: the intrinsic viscosity of the macromolecular gel is much greater than that of the low-molecular solvent, $\eta_{M}\gg \eta_{N}$.

To justify application of the Jeffreys scheme to the rotary motion of the particles (Figure 1), we remind readers that this phenomenology works rather well for the translational Brownian motion in semi-dilute polymer solutions. In such models [19,20], it is assumed that each particle is surrounded by a depletion layer where concentration of macromolecules is much lower than that in the bulk. Inside this thin layer, the viscosity is effectively small ($\eta_{N}$), and the diffusion process is fast (short times, small distances). However, when larger displacements (of the order of the particle size or greater) are considered, the particle experiences its environment as a medium with a high viscosity $\eta_{M}$. Similar behavior is inherent to biofilms [21], which, at short time intervals, respond as low viscous fluids but at longer scale react as weakly-fluent gels.

Following this line, we infer that a scheme with two rather different viscosity coefficients should be appropriate for the rotary diffusion of the particles furnished with a corona. Indeed, a loose macromolecular coating, just slightly changing the total mass of the system, at the same time strongly affects its rotary friction. Moreover, the presence of corona cannot be accounted for by just a simple renormalization of the particle hydrodynamic diameter since the corona brings in the particle dynamics a substantial retardation component. That is, if the particle turns over large angles, this motion entrains the whole corona. On the other hand, the corona is virtually insensitive to small angle displacements because such a motion concerns only the molecular fragments in close vicinity of the particle surface, and, due to that, the contribution of corona to resistance and retardation is insignificant. Therefore, at short time intervals, the particle may be considered as floating in a Newtonian fluid with low viscosity $\eta_{N}$.

For nanoparticles embedded in any viscous environment, the effect of inertia is negligible, and the process of rotary relaxation is always monotonic (overdamped regime). Given that, we chose as a main indicator of the state of corona the relaxation time of its elastic stress: $\tau_{M}=\eta_{M}/G$. In the theoretical consideration below, we analyze how the presence and magnitude of $\tau_{M}$ is reflected in the MRX spectra of the particle ensemble subjected to a bias DC field.

As in the adopted model, the magnetic moment $\vec{\mu }$ is “frozen” in the particle, and we take unit vector $\vec{e}=\vec{\mu }/\mu$ as a marker of the particle orientation. The equations of rotary motion for a Brownian particle in the inertialess limit take the form [17,18]:

(1) $$\dot{\vec{e}}=\left(\vec{\Omega }\times \vec{e}\right),\;\vec{\Omega }=\frac{1}{\zeta_{N}}\left[-\hat{\vec{L}}U+\vec{Q}+{\vec{y}}_{N}\left(t\right)\right],\;\hat{\vec{L}}=\left(\vec{e}\times \frac{\partial }{\partial \vec{e}}\right),$$

$$\left(1+\tau_{M}\frac{\partial }{\partial t}\right)\;\vec{Q}=-\zeta_{M}\vec{\Omega }+{\vec{y}}_{M}\left(t\right).$$

Here, $\vec{\Omega }$ is angular velocity of vector $\vec{e}$, and *U* is the orientation-dependent part of the particle energy, while $\hat{\vec{L}}$ is the operator of infinitesimal rotation with respect to $\vec{e}$. Vector $\vec{Q}$ in Equation (1) has the meaning of a torque acting on the particle on the part of the corona. The response coefficients of the viscoelastic medium are defined in a standard way [22] as

(2) $$\zeta_{\alpha }=6\eta_{\alpha }V,\;K=6GV,\;\alpha =N,\;M,$$

where *V* is the particle volume (we assume a sphere), and the subscript indicates either Newtonian (α=N) or Maxwell (α=M) viscosity (see Figure 1). With Notations (2), the stress relaxation time may be equivalently written as $\tau_{M}=\zeta_{M}/K$. We remark that there are no universal expressions to replace Formulas (2) provided the particles are non-spherical (anisometric). However, quite reliable estimates could be obtained if to approximate a particle with an ellipsoid of revolution (spheroid). For that shape, the effect of non-sphericity is rendered by a formfactor F that is to be inserted in Formulas (2) alongside $\eta_{\alpha }$ and *G*, respectively. The dependence of F as a function of the particle aspect ratio is known (see [23]) for an example.

Correlators of the random forces that model thermal noise in the system are expressed with the aid of the fluctuation–dissipation theorem:

(3) $$\langle y_{i\alpha }\left(t\right)\;y_{j\beta }\left(t+\tau \right)\rangle =2T\zeta_{\alpha }\delta_{\alpha \beta }\delta_{ij}\delta \left(\tau \right),\;\alpha =N,\;M;$$

note that, hereafter, we scale temperature in energy units.

The kinetic equation for the distribution function $W(\vec{e},\vec{Q},t)$ that corresponds to the set of stochastic Equation (1) is obtained via standard procedure (see [18,24,25]) for example:

(4) $$2\tau_{D}\frac{\partial }{\partial t}\;W=\left[\left(1+q^{-1}\right)\beta T\frac{\partial }{\partial \vec{Q}}-\hat{\vec{L}}\right]\left(\frac{1}{T}\vec{Q}+\beta T\frac{\partial }{\partial \vec{Q}}\right)\;W-\left(\beta T\frac{\partial }{\partial \vec{Q}}-\hat{\vec{L}}\right)\left[\frac{1}{T}\left(\hat{\vec{L}}U\right)+\hat{\vec{L}}\right]\;W.$$

Here, the following notations are used:

(5) $$\tau_{D}=\zeta_{N}/2T=3\eta_{N}V/T$$

for the Debye time of orientational diffusion of a spherical particle in a fluid of viscosity, and

(6) $$q=\zeta_{M}/\zeta_{N}=\eta_{M}/\eta_{N},\;\beta =K/T$$

for non-dimensional rheological parameters. The first one defines “maxwellity” of the Jeffreys medium, so that, at q=0, the model reduces to an ordinary viscous fluid; the second parameter is the dynamic elasticity coefficient scaled with thermal energy.

For a magnetically hard particle, the energy *U* that enters Equation (4) reduces to the Zeeman interaction with uniform field $\vec{H}$:

(7) U=−eH→H→=−μHeh→h→,h→2=1.

In non-dimensional form, the reference magnitude of that energy is

ξ=μH/T.

It is easy to verify that the equilibrium solution of Equation (4) is given by an extended Boltzmann distribution

(8) $$W_{0}\left(\vec{e},\vec{Q}\right)\propto \exp\left[-\frac{1}{T}\left(U+\frac{Q^{2}}{2K}\right)\right].$$

From Equation (8), it follows that the equilibrium state of the system in the absence of field U=0 is isotropic. Note that, although a constant external field endows the system with uniaxial anisotropy, the phase variables $\vec{e}$ and $\vec{Q}$ remain statistically independent in that case as well.

In a dilute (the interparticle interaction is neglected) statistical ensemble of such particles, the magnetization and susceptibility are rendered by expressions

(9) $$\vec{M}=n\mu \langle \vec{e}\rangle ,\;\chi_{ij}=\partial M_{i}/\partial H_{j},$$

where *n* is the number concentration of particles, whereas angular brackets denote averaging with the distribution function from Equation (4).

## 3. Dynamic Susceptibility

The MRX relaxation function, i.e., the dependence ΔM(H,t), where ΔM is the projection of magnetization on the direction of the probing field ${\vec{H}}_{1}$, and *H* is the bias field strength (see Figure 1), could be derived by several ways. We chose the one where the relaxation function is obtained in terms of the dynamic magnetic susceptibility χ(ω). The reason is two-fold. First, in the linear response approximation, the relation between the relaxation function and χ(ω) has a simple form. Second, in Refs. [18,26], we have developed a workable technique to obtain the pertinent dynamic susceptibility for the case of zero external field. Using those results, one may skip a considerable part of lengthy calculations and just add only the extension allowing for the bias field. Besides that, in [18,26], while analyzing the results obtained from solving the kinetic equation (the analogue of Equation (4)) in an exact way, i.e., using a long multi-moment expansion, we have shown that, to get a plausibly accurate approximation, it suffices to truncate the infinite moment set to just the first two equations for the dynamic variables δeH→=e→−〈e→〉0 and P→=Q→×eH→; here, angular brackets with index 0 denote averaging over the equilibrium distribution (8).

The aforementioned approximation is well known as the effective field model, and it has proven its usefulness for diverse problems of orientational kinetics of nanoparticles many times [12,27,28]. For the case of zero bias, the dynamic magnetic susceptibility in the effective field approximation is [28]:

(10) $$\tilde{\chi }\left(\omega ,\beta \right)=\frac{\chi (\omega ,\beta )}{\chi_{0}}=\frac{1}{1-i\omega \tau_{D}\left(1+\frac{\frac{1}{2}\beta }{1+\frac{1}{2}\beta /q-i\omega \tau_{D}}\right)}\;,$$

where $\chi_{0}=n\mu^{2}/3T$ is the static magnetic susceptibility of an ensemble of noninteracting particles bearing magnetic moments μ.

As seen from Equation (10), under zero bias the dynamic susceptibility is isotropic, and the type of its frequency dependence is determined by the elasticity β and “maxwellity” *q* of the medium (corona). At high temperatures β≪1, the viscoelastic properties of the corona do not manifest themselves, and the susceptibility reduces to a plain Debye formula with the reference relaxation time $\tau_{D}$. For a medium with high elasticity (β≫1), the dependence (10) has two maxima and may be with plausible accuracy presented as a superposition of two relaxation modes: slow (*s*) and fast (*f*) in the form

(11) $$\tilde{\chi }\left(\omega ,\beta \right)≃\frac{1-2/\beta }{1-i\omega \tau_{s}}+\frac{2/\beta }{1-i\omega \tau_{f}},\;\tau_{s}=\tau_{D}\left(1+\frac{\frac{1}{2}\beta }{1+\beta /2q}\right),\;\tau_{f}=\frac{2\tau_{D}}{\beta },\;\beta \gg 1.$$

Equation (11) shows that, under enhancement of elasticity, the slow component grows, and its peak moves further to the low-frequency domain, which means gradual increase of its reference time. Concurrently, the contribution of the fast relaxation mode goes down monotonically with β.

In the presence of bias field $\vec{H}$ that makes the system uniaxially anisotropic, the susceptibility becomes a second-rank tensor that is diagonal in the coordinate frame whose *Oz* axis points along the bias field:

$${\tilde{\chi }}_{ij}\left(\omega ,\beta ,\xi \right)={\tilde{\chi }}_{⊥}\delta_{ij}+\left({\tilde{\chi }}_{∥}-{\tilde{\chi }}_{⊥}\right)\;h_{i}h_{j}.$$

For the case of perturbation induced by a weak probing field ${\vec{H}}_{1}$ (in non-dimensional form, ${\vec{\xi }}_{1}$), the set of moment equations in the effective field approximation takes the form

(12) ∂∂t+γα(e)δet→α=12ξ→1fα+12βP→,

∂∂t+12β(1+q−1)+γα(M)P→=−12ξ→1fα+γα(e)δet→α,

with the coefficients

(13) $$\gamma_{⊥}^{\left(e\right)}=\gamma_{∥}^{\left(M\right)}=\left(1+c_{2}\right)/2\left(1-c_{2}\right),\;\gamma_{∥}^{\left(e\right)}=\left(1-c_{2}\right)/2\left(c_{2}-c_{1}^{2}\right),$$

$$\gamma_{⊥}^{\left(M\right)}=\left(3-c_{2}\right)/2\left(1+c_{2}\right),\;f_{⊥}=\frac{1}{2}\left(1+c_{2}\right),\;f_{∥}=1-c_{2},$$

defined in terms of functions

(14) $$c_{k}\left(\xi \right)=\int_{-1}^{1}x^{k}\exp\left(\xi x\right)dx/\int_{-1}^{1}\exp\left(\xi x\right)dx.$$

On solving Equations (12), one obtains the sought for dynamic susceptibility

(15) $${\tilde{\chi }}_{\alpha }\left(\omega ,\beta ,\xi \right)=\frac{\chi_{\alpha }\left(\omega ,\beta ,\xi \right)}{\chi_{0}}=\frac{1}{1-i\omega \frac{\tau_{D}}{\gamma_{\alpha }^{\left(e\right)}}\left(1+\frac{\frac{1}{2}\beta }{\gamma_{\alpha }^{\left(M\right)}+\beta /2q-i\omega \tau_{D}}\right)},$$

where index α assumes one of two possible values: ⊥ or ∥.

Presenting Function (15) as a sum of two relaxation modes—this time, for arbitrary values of parameters β and ξ and—one gets

(16) $${\tilde{\chi }}_{\alpha }\left(\omega ,\beta ,\xi \right)=\frac{A_{s}^{\left(\alpha \right)}\left(\beta ,\xi \right)}{1-i\omega \tau_{s}^{\left(\alpha \right)}}+\frac{A_{f}^{\left(\alpha \right)}\left(\beta ,\xi \right)}{1-i\omega \tau_{f}^{\left(\alpha \right)}},\;\tau_{s,f}=\frac{\tau_{D}}{\lambda_{s,f}^{\left(\alpha \right)}},$$

where the relaxation modes decrements $\lambda_{s,f}^{\left(\alpha \right)}$ are the roots of characteristic equation

(17) $$\lambda^{2}-\left[\Gamma_{\alpha }+\gamma_{\alpha }^{\left(e\right)}\right]\lambda +\gamma_{\alpha }^{\left(e\right)}\left(\Gamma_{\alpha }-\frac{1}{2}\beta \right)=0,$$

with $\Gamma_{\alpha }=\gamma_{\alpha }^{\left(M\right)}+\frac{1}{2}\beta \left(1+q^{-1}\right)$. The explicit expressions for the mode amplitudes and decrements are

(18) $$A_{s,f}^{\left(\alpha \right)}\left(\beta ,\xi \right)\;=\;\frac{1}{2}\;\;\left[1\pm \frac{\Gamma_{\alpha }-\gamma_{\alpha }^{\left(e\right)}}{\sqrt{{\left(\Gamma_{\alpha }-\gamma_{\alpha }^{\left(e\right)}\right)}^{2}+2\beta \gamma_{\alpha }^{\left(e\right)}}}\right]\;,\;\lambda_{s,f}^{\left(\alpha \right)}\;=\;\frac{1}{2}\left[\Gamma_{\alpha }+\gamma_{\alpha }^{\left(e\right)}\mp \sqrt{{\left(\Gamma_{\alpha }-\gamma_{\alpha }^{\left(e\right)}\right)}^{2}+2\beta \gamma_{\alpha }^{\left(e\right)}}\right]\;.$$

Let us consider the frequency dependence of the transverse (⊥) component of susceptibility (16). Under a weak constant field, it only slightly differs from the one rendered by Formula (10). However, in a medium with a pronounced elasticity, the fast mode might become dominating. Indeed, under a growing bias field, the decrement of the slow mode virtually does not change while $\lambda_{f}^{\left(⊥\right)}$ undergoes rapid increase, i.e., the relaxation time goes down. As a result, the spectrum as a whole shifts to the higher frequency range.

## 4. Magnetorelaxometry

### 4.1. Relaxation Functions

Consider an MRX experiment that is performed as follows. On the particle ensemble that dwells in equilibrium under a constant bias field $\vec{H}$, a weak perturbing field ${\vec{H}}_{1}$ is imposed transversely to $\vec{H}$. This induces the magnetization component ΔM=nμ〈δet→⊥〉 in the direction of ${\vec{H}}_{1}$. As soon as the magnetization attains its stationary value in the newly established equilibrium, the field ${\vec{H}}_{1}$ is turned off, and the process of relaxation of ΔM is recorded. This “transverse” configuration seems to be more preferable than measuring the longitudinal relaxation by varying $\vec{H}$ in small steps because the greater the relative magnitude of the relaxing perturbations (transverse vs. longitudinal), the stronger the bias.

According to the linear response theory (see [24], for example), the reaction to a stepwise turning off the field, is obtained from the dynamic susceptibility (15) by way of the integral relation

(19) $${\tilde{\chi }}_{⊥}\left(\omega ,\beta ,\xi \right)=1+i\omega \int_{0}^{\infty }e^{i\omega t}\;R_{⊥}\left(t\right)\;dt,$$

where $R_{⊥}\left(t\right)=\langle \delta e_{⊥}\rangle /{\langle \delta e_{⊥}\rangle }_{0}$ is the relaxation function, i.e., the transverse dynamic magnetization normalized by its initial value.

Subjecting Formula (19) to inverse Fourier transformation, one arrives at the expression for relaxation function

(20) R⊥(t)=2π∫0∞Imχ˜⊥(ω,β,ξ)ωcosωtdω.

As mentioned above, we take the stress relaxation time $\tau_{M}$ as the main characteristic of viscoelasticity of the macromolecular corona. If to describe the corona substance as a Jeffreys fluid, this time parameter is expressed in terms of the rheological coefficients as $\tau_{M}=\zeta_{M}/K=\eta_{M}/G$. Assuming that formation of the corona does not affect the viscosity $\eta_{N}$ of the solvent, one finds that, for presenting the relaxation function $R_{⊥}\left(t\right)$, it is convenient to scale the time in units of $\tau_{D}=3\eta_{N}V/T$. Moreover, the same quantity is appropriate as well for scaling the stress relaxation time $\tau_{M}$. On that basis, we introduce the non-dimensional parameter

(21) $$\kappa =\tau_{M}/\tau_{D}=2\zeta_{M}T/\zeta_{N}K=2q/\beta .$$

As seen, κ grows with the increase of “maxwellity” of the corona and/or of temperature and goes down with enhancement of the corona elasticity.

**Figure 2:**
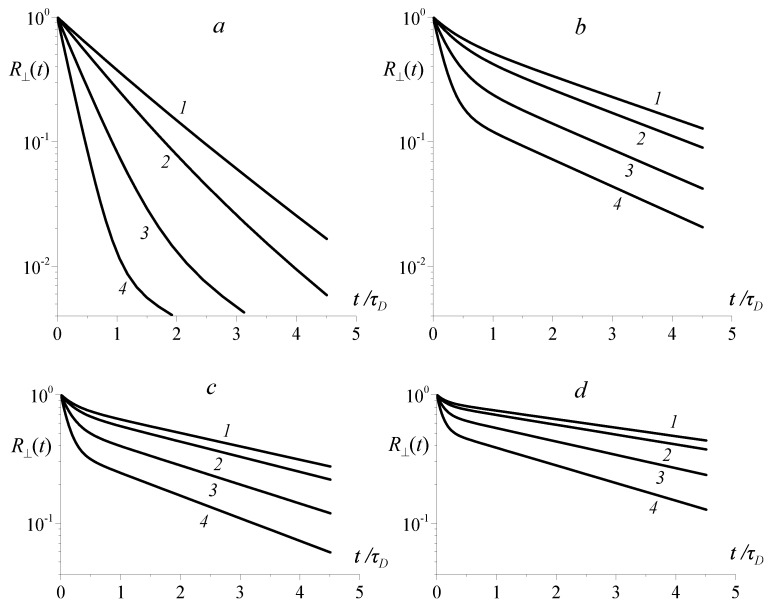
Relaxation functions for the “transverse” MRX; parameter $\kappa =2\times 10^{3}$ (**a**); 100 (**b**); 40 (**c**); 20 (**d**); the non-dimensional bias field is ξ=0.1 (*1*), 2 (*2*), 5 (*3*), 10 (*4*).

The plots illustrating the behavior of relaxation function $R_{⊥}\left(t\right)$ under variation of bias field *H* are presented in Figure 2. Their comparison reveals two main specific features. On the one hand, the more viscoelastic the corona, the slower the magnetic relaxation. On the other hand, the bias field, enhancing the effective restoring torque that acts on the particle, accelerates its orientational relaxation. Besides that, Figure 2 proves that magnetic relaxation, as soon as viscoelasticity (non-zero β) is introduced in the particle environment, is multi-mode, and formally the number of modes makes an infinite countable set. Meanwhile, in our model, this set is replaced just by two modes, slow and fast (see Equation (11)). However, as the detailed multi-mode analysis shows (would be presented elsewhere), this truncation does not affect the above-obtained essential qualitative conclusion: the considered relaxation process has two main reference time scales whose ranges differ by orders of magnitude. In logarithmic representation of Figure 2, the plot of $R_{⊥}\left(t\right)$ could be schematized as two quasi-straight lines—greater slope at short times and smaller slope at long times—connected by a crossover part. As seen from Figure 2—see curves *1* in all the panes—under zero bias, the crossover part is but weakly distinguishable. However, by using an appropriate bias field, the crossover part of $R_{⊥}\left(t\right)$ function could be enhanced and exposed to observation (see curves *4* in all the panes).

### 4.2. Integral Relaxation Time

For studying of multi-mode processes, a useful and experimentally easily determined characteristic is the so-called integral relaxation time that is defined as the area under the R(t) curve:

(22) $$\tau_{\alpha }^{\mathrm{int}}=\int_{0}^{\infty }R_{\alpha }\left(t\right)dt.$$

In the linear response approximation that we use here, the integral time expressed in terms of the dynamic susceptibility is

(23) ταint=limω→0Imχ˜α(ω,β,ξ)ω.

With Function (16), integral (23) is taken easily and yields

(24) $$\tau_{\alpha }^{\mathrm{int}}=\tau_{D}\frac{\Gamma_{\alpha }}{\gamma_{\alpha }^{\left(e\right)}\left(\Gamma_{\alpha }-\frac{1}{2}\beta \right)}=\tau_{D}\frac{\gamma_{\alpha }^{\left(M\right)}+\frac{1}{2}\beta \left(1+q^{-1}\right)}{\gamma_{\alpha }^{\left(e\right)}\left[\gamma_{\alpha }^{\left(M\right)}+\beta /2q\right]}.$$

This formula (with accuracy of the effective field approximation) in a qualitatively correct way describes the dependence of magnetization relaxation on all the relevant material parameters of the system. For example, for an isotropic system (ξ=0) assuming that β>1 and going back to dimensional time, one gets from Equation (23):

(25) $$\tau^{\mathrm{int}}=\tau_{D}\frac{1+\frac{1}{2}\beta \left(1+q^{-1}\right)}{1+\beta /2q}≃\tau_{D}\left\{\begin{array}{cc} 1+\frac{1}{2}\beta , & \beta \ll 2q,\\ 1+q,\; & \beta \gg 2q. \end{array}\right.$$

As the second line of Equation (25) shows, the asymptotic value of the integral time is defined by the slow diffusion. However, to really attain this regime, one needs a system with very strong elasticity: β≫2q. Even in dense polymer gels and “live polymers” where $q∼10^{3}-10^{5}$, one could hardly expect to match this condition. Under this limitation, one may always assume that the dependence of integral time (23) on the elasticity parameter is by and large linear.

In the presence of a bias field, full Expressions (13) for coefficients $\gamma_{\alpha }^{\left(e\right)}$ and $\gamma_{\alpha }^{\left(M\right)}$ should be used. Substitution of those in Equation (24) yields the formulas that enable one to estimate the effect of the bias field on the integral times of transverse and longitudinal relaxations of the dynamic magnetization:

(26) $$\tau_{⊥}^{\mathrm{int}}=2\tau_{D}\frac{1-c_{2}}{1+c_{2}}\left(1+\beta \frac{1+c_{2}}{3-c_{2}}\right),\;\tau_{∥}^{\mathrm{int}}=2\tau_{D}\frac{c_{2}-c_{1}^{2}}{1-c_{2}}\left(1+\beta \frac{1-c_{2}}{1+c_{2}}\right),\;\beta \ll 2q.$$

For a strong field (ξ≫1), Equation (26) expands to simple asymptotic expressions

(27) $$\tau_{⊥}^{\mathrm{int}}=\tau_{H}\left[1+\beta -\frac{2\beta }{\xi }\right],\;\tau_{∥}^{\mathrm{int}}=\frac{1}{2}\tau_{H}\left(1+\frac{1+\beta }{\xi }\right),\;\tau_{H}\equiv \frac{\zeta_{N}}{\mu H}=\frac{2\tau_{D}}{\xi }.$$

Therefore, as it follows from Equation (27), under a strong bias, the initial stage of relaxation is mostly due to the forced field-induced rotation of the particles with reference time $\tau_{⊥}^{\mathrm{int}}∼\tau_{H}∼2\tau_{D}/\xi$.

**Figure 3:**
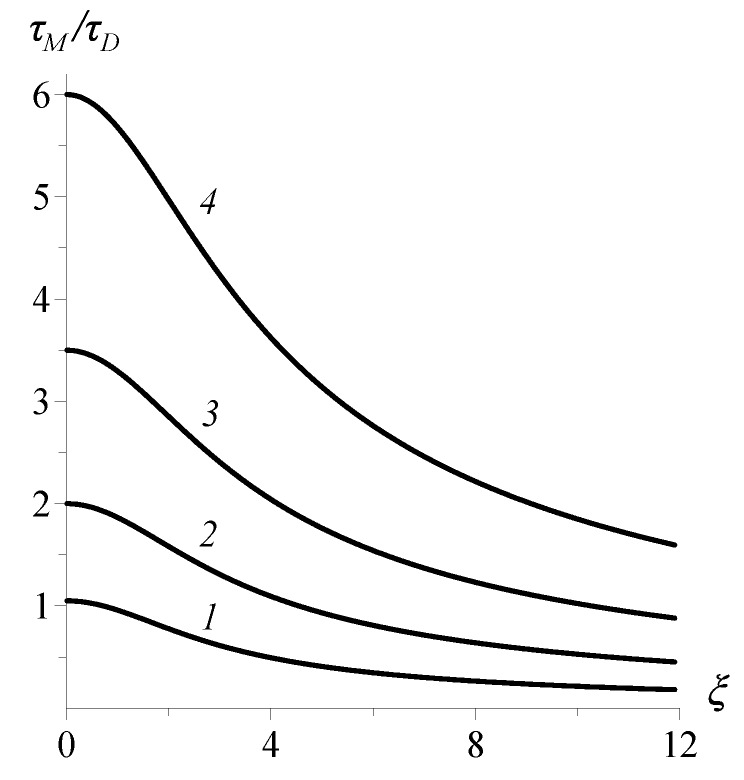
Dependence of the integral relaxation time on the strength of bias field for MRX in “transverse” configuration; the viscoelasticity parameter $\kappa =2\times 10^{3}$ (*1*), 100 (*2*), 40 (*3*), 20 (*4*); “maxwellity” of the corona is *q* = 100.

The overall behavior of the integral time is illustrated in Figure 3, where its dependence on the bias magnitude is given for different values of parameter $\kappa =\tau_{M}/\tau_{D}$. The values of integral time under zero bias obey very well Formula (25) for β≪q: the integral time grows linearly with the elasticity parameter β=K/T. As already mentioned, this increase might halt only at β≫2q, where $\tau_{⊥}^{\mathrm{int}}≃\left(q+1\right)\;\tau_{D}$, but that limit is practically unattainable. For the parameters used for plotting Figure 3, this upper limit would have ranged $\tau_{⊥}^{\mathrm{int}}/\tau_{D}≃100$.

## 5. Conclusions

To summarise the results of the work, three essential points should be mentioned. First, the macromolecular corona covering the particles is considered as a viscoelastic entity. This approximation is qualitatively different from a customary approach where the effect of corona is reduced to just a change of the particle hydrodynamic diameter. Second, we study MRX that is performed in the presence of a bias field, so that the bias field strength is a controllable parameter of the experiment. Third, the main attention is focused on the MRX measured in the “transverse” geometry where the probing field is applied normally to the bias one. The advantage of that variant comes from a significant enhancement of the response in comparison with that obtained in “longitudinal” configuration. Therefore, the transverse configuration enables one to preserve the sensitivity of the method despite that in general the bias field diminishes the particle response to orientational perturbations. We are well aware that the considerations presented here are to a certain extent illustrative as they are obtained with a severely simplified kinetic description: the whole set of moment equations has been replaced by just a pair of them (effective field model). However, our goal is to show, in the first place, the qualitatively relevant features of the considered problem. The work on a complete solution of the kinetic equation is under way.
